# Viewpoint of operating room nurses about factors associated with the occupational burnout: A qualitative study

**DOI:** 10.3389/fpsyg.2022.947189

**Published:** 2022-08-11

**Authors:** Esmaeil Teymoori, Armin Zareiyan, Saeed Babajani-Vafsi, Reza Laripour

**Affiliations:** ^1^Department of Surgical Technology, Faculty of Paramedical Sciences, AJA University of Medical Sciences, Tehran, Iran; ^2^Public Health Department, Health in Disaster and Emergencies Department, AJA University of Medical Sciences, Tehran, Iran; ^3^Social and Preventive Medicine Department, School of Medicine, AJA University of Medical Sciences, Tehran, Iran

**Keywords:** burnout, perioperative nursing, operating room, qualitative research, nursing

## Abstract

**Background:**

Occupational burnout is a mental health problem that among nurses may lead not only to physical and psychological complications, but also to a decrease in the quality of patient care. Considering the stressful nature of surgery, operating room nurses may be at a greater risk. Therefore, the present study aimed to identifying factors associated with the occupational burnout from the perspective of operating room nurses.

**Materials and methods:**

This qualitative study was conducted in Iran in 2021 using conventional content analysis. Sampling was performed using purposeful sampling method with maximum variation. In order to collect data, individual, semi-structured interviews were performed with 18 operating room nurses. Interviews continued until data saturation. Data were recorded, transcribed and analyzed using steps proposed by Graneheim and Lundman. Data management was performed using MAXQDA-2020 software. Four criteria provided by Guba and Lincoln were used to improve the study’s trustworthiness and rigor.

**Results:**

A main theme, 4 categories and 15 subcategories were extracted from the data. The main theme is “gradual burnout due to job tension” and categories and subcategories include organizational factors (manager incompetence, organizational indifference, ambiguity in organizational role, organizational inconsistency), interpersonal factors (surgeon aggression, surgeon authoritarianism, surgeon failure to manage stress, unprofessional behavior of nurses), Occupational nature factors (psychological factors and occupational hazards) as well as individual factors (occupational attitude, unprofessional behavior, emotional involvement, demographic factors, physical factors).

**Conclusion:**

Numerous factors associated with burnout in operating room nurses, which may put the personnel under more pressure. According to these factors, nursing managers and operating room nurses can consider effective strategies to prevent or coping with burnout.

## Introduction

Occupational burnout is a mental health problem, which is classically defined as a long-term response to work-related stress and is associated with reduced personal accomplishment or ineffectiveness, depersonalization or cynicism, as well as emotional exhaustion in the workplace (Maslach and Leiter, 2016). In the late 1960s, the concept was used in health care organizations to describe the emotional stress among health care professionals (H). It can be intensified by a number of factors, including high workload, unhealthy work environment, and lack of organizational support (Freudenberger, 1974; Alrawashdeh et al., 2021). Previous studies have shown factors such as stressful work environment, inadequate staffing (Shah et al., 2021) long working hours, job dissatisfaction (Meng et al., 2021) inadequate supply of equipment and human resources, ineffective managerial approaches (Ghavidel et al., 2019), economic conditions and lack of motivation (Kanaani Azari et al., 2020) are related to the burnout of health care professionals.

Occupational burnout is a health threat to health care professionals (HCPs) and can lead to depression, increased risk of suicide, personal dissatisfaction, and compromised patient care (LaFaver et al., 2018; Agarwal et al., 2020). HCPs prone to burnout may be less responsive to patient needs while patient care requires extreme caution and a small mistake can lead to serious risks (Devery et al., 2018; Shahzad et al., 2019). Among all occupations, nurses are more prone to burnout due to their unique interaction with the patient, which is a major concern given the nature of their job (Gómez-Urquiza et al., 2017; Phillips, 2020). More than 50% of the nursing population in the United States and around the world, one in ten nurses experience burnout (Jun et al., 2021).

Nurses are expected to provide high-quality, evidence-based treatment in high-tech workplaces with significant patient turnover (Kowalczuk et al., 2020). Therefore, paying attention to their mental health, which also affects the rapid recovery of patients, is vitally important. Studies have shown that burnout in nurses is affected by heavy workload, co-workers’ behavior, environmental factors, organizational factors, and the nature of diseases that directly affect their job performance (Shahzad et al., 2019) to the extent that it can lead to job turnover and intention to leave job. The shortfall of nurses is predicted to reach 260,000 by 2025, so it is essential to pay attention to mental health and improve the work environment (Phillips, 2020). Of course, it should be noted that burnout is a reaction to stressful events and how each person reacts to such situations is determined by the way he analyzes it (Koutsimani et al., 2019).

The high stress and burnout of nurses has become a global challenge (Shahzad et al., 2019). Burnout is seven times more likely to occur in high-stress environments than in low-stress areas (Kacem et al., 2020). The operating room is a unique department that can create more stress due to its special circumstances. It has a high level of stress and complexity and the organization expects not even the smallest mistake in this environment (Aslani et al., 2019; Teymoori et al., 2022). As a member of the surgical team, operating room nurses are involved in patient admission, surgical intervention and patient care in the operating room. Their performance guarantees the safety of the surgical patient and the quality of care (Sillero and Zabalegui, 2018; Nahid et al., 2021).

During complex surgeries, operating room nurses have to work long hours with high concentration, constant attention to patient safety and in night shifts in emergency surgeries. So, they are exposed to high physical and psychological tension (Li et al., 2021). In addition, they are faced hazardous objects including chemicals and disinfectants, radiographic rays, sharp objects and blood pathogens, surgical smoke, waste anesthetic gases, long sleep deprivation, injuries caused by long standing and holding the instruments and equipment during surgery. In other words, these stressful problems include biological, chemical, and physical factors that may increase burnout (Rothrock, 2019; Li et al., 2021; Fereidouni et al., 2022).

In a study of more than 50,000 American nurses, researchers found that burnout was the leading cause of 74.9% of people leaving their jobs or intending to leave job (Shah et al., 2021). Although most burnout studies have focused on general nurses and only a few have investigated operating room nurses (Li et al., 2021), the results of a study by Sillero and Zabalegui in Spain showed that operating room nurses suffer from moderate to high burnout. The researchers also stated that the operating room is an unfavorable working environment for nurses and organizational factors play an important role in burnout (Sillero and Zabalegui, 2018). In their study, Almodibeg and Smith stated that although the prevalence of burnout was low among operating room nurses, these individuals were at a high risk. Numerous factors such as high workload, insufficient salary, job risks and lack of organizational support were cited as the main causes of burnout (Almodibeg and Smith, 2021). Limited studies on burnout of operating room nurses were found in Iran. The results of a study by Mirmortazavi and Ghafari showed that 47.8% of Iranian operating room nurses experience burnout and are severely involved in restlessness, physical problems, and depersonalization phenomenon that the issue requires special attention (Mirmortazavi and Ghafari, 2017). Previous studies have mostly investigated the burnout of nurses in different departments of the hospital in a descriptive and quantitative method (Medeiros-Costa et al., 2017; Almodibeg and Smith, 2021; Li et al., 2021). While conducting qualitative studies is necessary to better understand the concept of job burnout among nurses (Medeiros-Costa et al., 2017). Qualitative study provides researchers with deep insight into participants’ understandings and experiences. The goal of the qualitative researcher is to collect details and understand human behavior about the subject under investigation in the context and culture of the people themselves (Salaree et al., 2021). Considering the vital role of operating room nurses in surgery, the abstractness of the concept of burnout and the lack of knowledge of its related factors among operating room nurses, also depending on the context of this concept in different societies, the researchers decided to conduct this study in order to provide a better understanding of burnout based on the experiences of operating room nurses. This qualitative study was conducted with the aim of identifying factors associated with the occupational burnout from the perspective of Iranian operating room nurses. Using the results of this study, organizational managers and operating room nurses could identify the factors of occupational burnout and develop and implement appropriate strategies to prevent and coping with it.

## Materials and methods

### Study design

This is a qualitative study with conventional content analysis approach. Qualitative study can provide the researcher with a deep insight into the perceptions and experiences of individuals. In content analysis, concepts are extracted directly through interviews or other methods of data collection. In this method, after coding the data, subcategories, categories and themes can be identified in the text (Hsieh and Shannon, 2005). This study was conducted between January 2021 and February 2022 in Tehran, Iran. This study is designed based on COREQ (Consolidated criteria for reporting qualitative research) guideline (Tong et al., 2007).

### Study population/sampling

Participants included 18 operating room nurses from hospitals of Tehran (Imam Reza, Besat, Imam Khomeini, Firoozgar, Talaghani, Baqiyatallah, and Rasoul Akram). Purposive sampling method with maximum variation was used for sampling (in the age, gender, work experience, and work shifts of participants), it was done by the main researcher (The first author of the article) under the supervision of the research team. Purposive sampling is useful in qualitative research because the researcher investigates the subject in participants who have experienced it (Rozo et al., 2017). The main researcher selected individuals who satisfied the inclusion requirements. Inclusion criteria were at least 1 year of work experience in the operating room, full-time employment in the operating room, and the willingness of individuals to share their experiences on the subject of this study. Also refusing to record and uncompleted interview were considered as the exclusion criteria.

### Data collection

The interview was conducted by the main researcher of the study who was a faculty member of operating room department at the time of the study. The main researcher participated in training sessions on qualitative research methods and interviews and successfully completed it. Before starting the interview process, two pilot interviews were conducted under the supervision of professors who were experts in qualitative research to check whether the interview process can extract the required data from the participants. In order to collect data, individual, face-to-face, semi-structured interviews were used. The duration of the interview varied from 40 to 80 min and some participants were re-interviewed for completion of information. In the re-interview, the participants were asked to further explain the ambiguity in the answers to the questions for the researcher. The interviews continued until data saturation, i.e., until new data would not lead to the provision of new information (Marshall et al., 2013). Although data saturation was achieved from the 15th interview, up to 18 interviews were conducted for higher confidence. Prior to the interview, the purpose of the study was explained and informed consent was obtained from the participants. As set out by the participants, the time and place of the interview conducted was determined at their workplace (personnel rest room in the operating room) and in a calm environment. All interviews were recorded in entirety. The interview started with general triggers such as: “What were your experiences of stressful days in the operating room” and continued with probing questions such as: “Please explain more,” “What do you mean by that?” Or “Can you give an example?” based on previous responses in order to clarify the details, ambiguities and increase the depth of the interviews. Before the first interview, the interview question was prepared and approved by all the authors during several discussion sessions. Table 1 provides the interview question guide.

**TABLE 1:** Interview question guide.

Type of questions	Example of questions
Introduction	Introduction of the main researcher Explaining the objectives and importance of the study Voluntary participation in the study Use of data anonymously No right or wrong answer and explain your opinion in every question Obtaining informed consent
Beginning	What were your experiences of stressful days in the operating room? What situations make you endure more stress? What are the things in the operating room that may harm you? What problems and injuries are associated with working in the operating room? What things can be related to burnout of operating room nurses? What makes you feel most fatigued and exhausted about your work? Are environmental factors such as working conditions, colleagues’ behavior involved in stress and burnout? Further questions based on the objectives of the study and answers of the participants.
Development	Including probing questions Please explain more about this subject. What do you mean by that? Can you give an example? What do you think is the reason for this problem?
Closing	What would you like me to ask related to the burnout of operating room nurses? Are there any untold things about the burnout of operating room nurses?

### Data analysis

The process of interviews and data analysis was performed simultaneously with conventional content analysis. Data analysis was performed using the steps proposed by Graneheim and Lundman (2004). According to this method, by the main researcher the content of each recorded interview was transcribed verbatim and the interviews were read line by line several times to gain a general understanding of the text. The transcription of the interview was then divided into small sections, each of which provided a specific meaning and concept as meaning units, to which code or codes were assigned based on the concept. Then, by constant comparison of each code with other similar codes, the same codes were classified as subcategories. A list of the subcategories was then obtained. Based on similarities, differences and relationships of the subcategories with each other, categories were classified and finally the theme was extracted. The coding process of transcribed interviews was facilitated using MAXQDA-2020 software.

### Trustworthiness and rigor

In the present study, four criteria (credibility, dependability, confirmability, and transferability) were used to ensure the trustworthiness and reliability of the data (Lincoln and Guba, 1986). To achieve credibility, long-term involvement in data and triangulation was considered. The triangulation of data included considering the maximum variation in the age and gender of the participants, the time of the interview in various work shifts, the place of the interview in various operating room departments and hospitals, as well as the use of member check and peer check (Cochran and Elder, 2014; Nahid et al., 2021). Interview transcriptions were returned to the participants to determine if they meant what was written (Member Check). In addition, the opinions of the research team professors that expert in qualitative study, regarding the process of conducting interviews, the analysis and extracted data were used (Peer Check) which provided the dependability of the study. For dependability of the data the opinions of all the research team were used in the analysis and coding. In order to conformability, the all the extracted codes, categories, and sub-categories and parts of the interview text were reviewed and confirmed by three researchers who were outside the research team and were familiar with the qualitative method. Also, the coding process was tried not to be influenced by the opinions of the researchers. For minimize the bias on behalf of the researchers, reflexivity was used in all steps of the study. For transferability of the data, direct quotes from operating room nurses as well as comprehensive explanations of research steps, characteristics of participants, data collection and analysis were provided.

### Ethical consideration

After approval by the research vice chancellor of the relevant university, the permit of this study was approved by the ethics committee of Aja University of Medical Sciences (with the ethics code IR.AJAUMS.REC.1399.277). At the beginning of the interview, the researcher, after introducing himself, explained the objectives and nature of the research to the participants and allowed them to complete or stop the interview. Also provided informed oral and written consent from the samples. All participants were assured that their information is confidential so that the data will only be shared with the research team and the interviews replaced with codes so that individuals could not be identified. In addition, they were explained that they had the right to withdraw from the study at any stage.

## Results

Participants in this study included 18 operating room nurses (10 females and 8 males). The age of the participants was 26 to 46 (34.33 ± 5.86) with a work experience of 3 to 24 years (11.16 ± 6.42) (Table 2). The results of data analysis in this study revealed the factors associated with the occupational burnout of operating room nurses. These factors included a main theme, 4 categories and 15 subcategories (Table 3).

**TABLE 2:** Demographic characteristics of the participants.

Variable	Category	Number	Percentage
Gender	Female	10	44.4
	Male	8	55.6
Marital status	Single	6	33.3
	Married	12	66.7
Education level	Associate degree	2	11.1
	Bachelor’s degree	11	61.1
	Master’s degree	5	27.8
Shift work	Fixed	8	44.4
	Variable	10	55.6
Type of employment	Permanent	15	83.3
	Contractual	3	16.7
Working in another hospital	Yes	9	50
	No	9	50
Managerial position	Yes	7	38.9
	No	11	61.1
Hospital type	Governmental	14	77.8
	Non-Governmental	4	22.2

**TABLE 3:** Theme, categories, and subcategories.

Theme	Categories	Subcategories
Gradual burnout due to job tension	Organizational factors	Manager incompetence
		Organizational indifference
		Organizational inconsistency
		Ambiguity in organizational role
	Interpersonal factors	Surgeon’s aggression
		Surgeon’s authoritarianism
		Surgeon’s failure to manage stress
		Unprofessional behavior of nurses
	Occupational nature factors	Psychological factors
		Occupational hazards
	Personal factors	Occupational attitude
		Unprofessional behavior
		Emotional involvement
		Demographic factors
		Physical factors

### Theme: Gradual burnout due to job tension

By analyzing the data from the study, the burnout factors of operating room nurses were obtained in four categories “organizational factors, interpersonal factors, Occupational nature factors, and individual factors.”

### Organizational factors

Findings show that one of the main concerns of most participants about burnout is related to the organization, in such a way that operating room nurses said that hospital managers have created additional workload with their decisions and performance. This category includes four subcategories: “manager incompetence, organizational indifference, organizational inconsistency, and ambiguity in organizational role.”

#### Manager incompetence

Participants stated that one of the main reasons for burnout in the operating room was the poor performance of the head nurse and other hospital managers. They referred to “creating discrimination by the manager.” In most cases, it was pointed out that the work is not divided among all nurses and people have to endure more work pressure. In addition, the division of responsibilities in the organization is not based on competence. The operating room nurses also stated other characteristics of the manager, such as “inability to manage, irresponsibility, lack of criticism, and lack of support” as factors that can cause tension and burnout in the operating room environment.

*“When he head nurse gives his nurse friend more rest than me, and I have to work more, it’s annoying. I expect from the operating room head nurse to support me when the surgeon talks unjustly, but that doesn’t exist. She takes side with the surgeon in any situation.” (Participant No. 9, woman with 18 years of work experience)*

#### Organizational indifference

According to the participants, “not appreciating the quality of nurses’ work, manager’s inattention to lack or defective of equipment, low salaries and benefits, shortfall of nurses, and inappropriate physical facilities” are some of the things that can cause double pressure on operating room nurses.

*“I try to work my best in the operating room according to my experience, but I am not seen at all and it does not matter. This lack of attention is reflected in the reports to the higher manager and their evaluations. In addition, there is no correlation between salary and the work done.” (Participant No. 11, woman with 10 years of work experience)*

#### Organizational inconsistency

Operating room nurses stated that there is no proper coordination between the different departments of the operating room and the hospital, that’s why the work schedule of the personnel may be affected. They stated “lack of coordination between different hospital departments and the sudden change of shifts” as factors that can lead to loss of peace of mind and concentration.

*“Sometimes we ask the CSR department for a surgical set, but it is not ready. This confuses us about the exact time the surgery will start*…. *One day I had to be on shift in the evening, but the head nurse the last night sent a message that the number of staff was not enough and you had to come on shift in the morning. It ruined my whole life plan that day.” (Participant No. 6, woman with 7 years of work experience)*

#### Ambiguity in the organizational role

According to the comments of the participants in the study, the ambiguity in the task leads to the confusion of nurses and can prevent them from focusing on their work. Some nurses stated “unspecified tasks in the operating room and working more than tasks” as factors in burnout.

*“As a scrub person in the operating room, I just have to transfer the surgical instruments to the surgical team, but I also have to play the role of the first surgeon’s assistant and work more. It is not clear what our duties are in the operating room.” (Participant No. 7, woman with 3 years of work experience)*

### Interpersonal factors

According to participants, factors related to surgeons and other operating room nurses can lead to burnout. This category consists of four subcategories: “surgeon’s aggression, surgeon’s authoritarianism, surgeon’s failure to manage stress, and nurses’s unprofessional behavior.”

#### Surgeon’s aggression

Participants reported the surgeon’s violent reactions, including “shouting, insulting, getting angry, and humiliating.” They said these cases could lead to distraction and reduced job quality.

*“Some surgeons may insult due to smallest causes, especially if the situation is stressful. When the surgeon gets angry, it causes me to act hastily and even an error may occur.” (Participant No. 8, woman with 6 years of work experience)*

#### Surgeon’s authoritarianism

Some nurses described the operating room climate as having a “physician dominance atmosphere.” They stated that there is viewpoint of “Self-aggrandizement by surgeon” and complained about “surgeon dominance” in the operating room. In such an environment, nurses may feel inferior and find his/her work worthless.

*“The hospital managers cares about the physician more than any other medical profession. In the operating room, unfortunately, some surgeons think that because they have a higher educational degree, all of their orders should be fulfilled, and they consider themselves entitled in any situation.” (Participant No. 1, man with 13 years of work experience)*

#### Surgeon’s failure to manage stress

Some participants stated that some surgeons are not able to manage their stress when the surgery becomes complicated. Operating room nurses referred to problems such as “surgeon anxiety, transmit stress to the surgical team and misbehavior due to stress such as Throwing surgical instruments or leaving the patient.”

*“During surgery, if the patient bleeds or has any problem, the surgeon immediately gets a lot of stress. He may even leave the patient or throw a surgical instrument. This is a terrible situation.” (Participant No. 10, woman with 6 years of work experience)*

#### Nurses’s unprofessional behavior

Participants mentioned factors related to the behavior of other nurses in the operating room environment. These included “lack of empathy and friendship, lack of proper collaboration, slander, selfishness, jealousy, flattery, hypocrisy, and demotivation.”

*“There is no good collaboration and friendship among operating room nurses. Some co-workers may treat me kindly at this moment but the next day they slander me in the presence of the manager or someone else. That’s unbearable. Some colleagues only work for their own benefit.” (Participant No. 11, woman with 10 years of work experience)*

### Occupational nature factors

According to the participants, some factors related to the nature of the job can cause fatigue and burnout. This category consists of two subcategories “psychological factors and occupational hazards.”

#### Psychological factors

Some of the nurses participating in the study included items such as “high stress in the operating room, unpredictable events in some complex surgeries, working in closed environments, night shift work pressure, high workload and require for high concentration during surgery” as factors associated with burnout.

*“The operating room is a stressful environment, especially if the surgery goes against the plan. This amount of hard work is really overwhelming.” (Participant No. 12, woman with 8 years of work experience)*

#### Occupational hazards

The experiences of some participants showed the “physical, chemical, and biological occupational injuries” in the operating room. Physical injuries can include foot pain due to long standing, back pain due to moving heavy equipment, wrist pain due to long-term holding of surgical instruments. Chemical injuries such as inhalation of disinfectants and anesthetic gases, as well as biological injuries including the transmission of pathogenic microorganisms from the patient, needle stick injuries and surgical smoke can be mentioned.

*“As a student, I couldn’t believe that young staff would have varicose veins, but I have had this complication in the few years I have been working. My friends had a needle stick during surgery, and unfortunately the patient had hepatitis. She has been testing and taking various drugs for a long time.” (Participant No. 14, woman with 3 years of work experience)*

### Personal factors

Participants identified some personal factors as the cause of burnout. This category includes five subcategories: “occupational attitudes, unprofessional behavior, emotional involvement, demographic factors, and physical factors.”

#### Occupational attitude

Some nurses said that although the operating room is one of the most important parts of the hospital, the nurses of this department are not given enough attention. According to the interviews, “lack of job motivation, job dissatisfaction, and indifference to the job” were among the reasons that operating room nurses did not enjoy their work and had to endure it.

*“The operating room environment does not appeal to me at all. I feel useless when I am constantly doing repetitive tasks. I do not see any progress, even if I do my best.” (Participant No. 5, woman with 11 years of work experience)*

#### Unprofessional behavior

Some of the nurses participating in the study stated factors such as “comparing their conditions with surgeon and nurses of other departments and distrusting to colleagues” as factors that can cause burnout.

*“If I go back to the past, I will definitely try harder to be a surgeon because there is a high salary gap between nurses and the surgeon. People pay more attention to surgeons.” (Participant No. 8, woman with 6 years of work experience)*

#### Emotional involvement

Some participants stated that in situations such as “patient’s death in the operating room, and patients with emergency condition such as trauma” affect them very much and they could not function properly for several days after this event. Also “Feeling of inadequacy in providing services to patients in the end stage of disease and feelings of routine in operating room” were other causes of burnout.

*“It’s really hard when I watch the patient die in operating room. I remember talking to a patient before the surgery but he died during the surgery and I couldn’t do anything. I was not well for several days after this event.” (Participant No. 4, man with 18 years of work experience)*

#### Demographic factors

Some participants stated that people with less experience have to work more so that in addition to their duties, they have to perform the duties of colleagues with more experience. Also, according to the opinion of the nurses participating in the study, married personnel should work more than single personnel due to economic conditions. Some nurses stated that “people with lower work experience, women as well as married ones” endure more work pressure.

*“When I protested why I should work harder while my colleagues rest, they said because you have less work experience. In the operating room, everything is for the benefit of someone with higher experience.” (Participant No. 7, woman with 3 years of work experience)*

#### Physical factors

Participants cited “fatigue from working in more than one hospital, moving heavy objects, standing for long periods of time during surgery, as well as conflict of work and family lives” as factors associated with burnout.

*“I was on a night shift at another hospital last night and I’m at this hospital this morning because the income and expenses are not adequate. I can say that most people who work here are in the same situation and it definitely makes us feel more tired.” (No. 2, man with 19 years of work experience)*

## Discussion

This qualitative study was conducted to identify the factors associated with the Occupational Burnout from the perspective of Iranian operating room nurses. The present study showed that several factors cause burnout. This is the first qualitative study on the causes of burnout in operating room nurses in Iran. The analysis of participants’ experiences led to the extraction of a theme called “gradual burnout due to job tension” and four categories including “organizational factors, interpersonal factors, Occupational nature factors, and personal factors.”

One of the most important causes of burnout in operating room nurses is the poor performance of the organization. The operating room is one of the high-risk environments where any mistake can have irreparable consequences (Aslani et al., 2019). Due to the difficult conditions of this environment, proper support of the organization can make the operating room nurses more motivated in line with the goals of the organization and have a good performance. Hospital managers must provide equal conditions for all staff so that people do not feel discriminated in the workplace. In the operating room, only the abilities and competence of the nurses should be considered as the basis for evaluation, career promotion or division of responsibilities. Improper management and discrimination are important problems that have been considered as effective factors in burnout of operating room nurses in the present as well as other studies (Sillero and Zabalegui, 2018; Imani et al., 2022). The findings showed that operating room nurses expects that their services and quality of their work are appreciated by managers in special operating room conditions. When an organization does not Attention to the performance of its employees, their enthusiasm may gradually have lost and the quality of services provided may be affected.

Consistent with the present study, studies conducted by Almodibeg and Smith (2021); Nahid et al. (2021), and Imani et al. (2022) have emphasized the role of factors such as lack of appreciation and inadequate pay, shortfall of nurses, lack or inadequate equipment, and inappropriate physical facilities in operating room nurses burnout. Ambiguity in the duties of operating room nurses was another issue that was expressed by study participants as a cause of burnout. The operating room nurses participating in the present study stated that when they are scrub role, they should also perform tasks of first surgeon’s assistant role. Unclear job descriptions can confuse nurses and make them feel that this is not their duty and has been imposed on them. This may be due to the nurses’ lack of awareness or the organization’s compulsion. In line with the present study, previous studies in the United States and Turkey also pointed to nurses’ dissatisfaction with role ambiguity, which can lead to work stress and ultimately more burnout (Wooldridge et al., 2020; Sanli et al., 2021). Participants reported inappropriate behaviors of some surgeons in the form of shouting, insulting, anger, humiliation, as well as the surgeon’s dominance and his failure in stress management among the causes of burnout in the operating room. In line with the present study, previous studies have also focused on this type of surgeon behavior and referred to it as “disruptive physicians,” which can be verbal or physical and ultimately have a negative impact on nurses and patients (Cochran and Elder, 2015).

One of the reasons that the operating room can be considered as a special department from other department of the hospital is the direct communication between the surgeon and the nurse in a very stressful environment where all the focus should be on the surgical procedure. In such situations, the lack of proper communication between people can lead to failure in the team activities that required for surgery and can endanger the lives of patients and operating room staff. Poor teamwork has been considered as an effective factor in reducing the resilience and increased burnout of operating room nurses (Nahid et al., 2021).

Consistent with the results of the present study, a study conducted by Cochran and Elder in the United States showed that the surgeon, due to his dominance in the operating room, shows his most severe destructive behaviors toward those who have the least power in this environment. In this situation, operating room nurses are more vulnerable. This can lead to increased burnout. The behavior of surgeons is possible due to the high revenues the surgeon makes for the hospital, ignored by organizational managers and therefore do not have bad consequences for them. This issue has also been mentioned in previous studies (Cochran and Elder, 2014). In addition, participants cited the unprofessional behaviors of other operating room nurses as factors of burnout. Most of the mentioned cases were related to lack of empathy and friendship, lack of proper collaboration, slandering others, selfishness and jealousy. Previous studies showed that inappropriate relationships between nurses in the workplace significantly affect their mental health. When nurses had better relationships at work, they had significantly lower scores on fatigue, stress, and burnout (Wei et al., 2018). In line with the results of the present study, the conducted research also introduces social climate as one of the factors of nurses’ burnout, which includes the interpersonal relationships of nurses with each other or with superiors at work (Pérez-Fuentes et al., 2018).

The operating room is one of the most complex environments in the health care system, with many inherent risks to the surgical team (Simon et al., 2016). High stress and workload, night shift pressure, unpredictable events in some complex surgeries, working in closed environments and occupational hazards were among the items cited by participants as causes of burnout. Previous studies have shown that the nature of surgery causes operating room nurses to be exposed to physical, emotional and psychological fatigue. Burnout is one of the main factors in reducing the resilience of the operating room nurse, which is due to job stress, night shift and high working pressure (Nahid et al., 2021). In addition, the occupational hazards mentioned included physical injuries (such as low back pain, X-rays), chemical injuries (such as disinfectants and sterilizers), and biological injuries (such as exposure to infectious agents and surgical smoke). Previous studies have shown that 84% of operating room nurses are affected by this type of risk (Saleh et al., 2020). Occupational hazards can affect people’s performance and cause burnout (Somville et al., 2016).

Some participants expressed job dissatisfaction, lack of motivation and feelings of routine in operating room as factors influencing burnout. Operating room nurses can be effective when they are interested in their job. Previous studies emphasize that job satisfaction and burnout affect each other and the level of job satisfaction can be used as a predictable indicator of burnout in nurses (Cilingir et al., 2012). Decreased job satisfaction and motivation can negatively affect the physical, mental and social health of employees, reduce the quality of work and lead people to leave their jobs. Operating room nurses spend a lot of time in an environment (operating room) with very little contact with the outside world. These conditions, along with the heavy workload, can make people feel tired. Therefore, workplace attractiveness and organizational support can be considered as factors to increase people’s motivation to work (Imani et al., 2022). According to the obtained results, nurses stated that women and married people are more exposed to job burnout. Contrary to the present study, the participants stated that women experience more work pressure and subsequently more burnout, but a previous study in Spain showed that the risk of burnout is higher in men (Pérez-Fuentes et al., 2019), which may be due to differences in the context of societies. Also in line with the present study, the conducted research about burnout of Iranian nurses showed that burnout is more in married personnel than unmarried ones (Matin et al., 2014).

According to the results, some operating room nurses with items such as unresolved conflicts between work and family, Lack of ergonomic principles, constantly comparing their income with surgeons or nurses of other departments, distrusting to colleagues and working simultaneously in more than one hospital, expose themselves to further burnout. Previous studies have shown that workplace stress can lead to inappropriate or unprofessional employee reactions. Unprofessional behavior has a negative effect on employee-workplace relationships and can cause more fatigue (Parizad et al., 2018). Previous studies have also shown that work conditions such as fatigue and stress in the workplace affect nurses’ personal lives. As an employee and a member of the family, nurses must play both roles simultaneously. These incompatible reciprocal roles highly affect burnout (Shahzad et al., 2019; Mahvar et al., 2020). According to the results, nurses stated that due to economic conditions, they have to work more hours or work in more than one hospital. Studies have shown that there is a significant and negative relationship between job burnout and the economic conditions of staff, so that personnel with low economic conditions experience more burnout (Matin et al., 2014; Irandoost et al., 2021). Also, neglect of ergonomic principles among operating room nurses in cases such as prolonged standing and moving heavy objects has led to musculoskeletal disorders and burnout (Nahid et al., 2021).

### Limitations

The present study has a number of limitations. The findings of the present study were obtained from hospitals in the capital of Iran, which generally has larger and more equipped hospitals than other cities, and may differ in other areas according to workload, organizational support, interpersonal relationships, and operating room characteristics, so may not be generalizable. Participants may have not disclosed all the information due to considerations such as fear of measures taken by organization’s managers or surgeon as a result of the interviews that the researchers tried to reduce this limitation with cases like individual interviews and assuring participants that information will not be disclosed. Furthermore, although the study results revealed burnout factors, they might not be generalizable due to the qualitative nature of the research. Also, although the interviews were held during the personnel’s break time and in a quiet place, the obtained data may have been affected due to fatigue caused by workload or lack of concentration. In addition, the participants may have a bad feeling toward the managers of the organization or their work at a certain point of time, and the information obtained is due to it that the researchers tried to reduce the role of this limitation by conducting interviews over a long period of time.

## Conclusion

Previous theories stated the factors of emotional exhaustion, depersonalization, and diminished sense of personal accomplishment related to job burnout. But this study was conducted in order to investigate this issue in the specific context of Iranian operating room nurses. Findings of this study showed that Iranian operating room nurses are faced with several factors of burnout including organizational, interpersonal, occupational nature factors, and personal factors. Participants stated inadequate support of the organization in various dimensions, inappropriate behavior of surgeons and other operating room nurses, factors related to the nursing job in the operating room and personal factors as factors of burnout. According to these factors, nursing managers and operating room nurses can consider effective strategies to prevent or coping with burnout. The goal of these measures is to reduce the negative effects of burnout on operating room nurses and ultimately improve the quality of care. For example, senior nursing managers can help reduce burnout by choosing competent managers, providing sufficient number of nurses, matching benefits and salaries with work, and creating job motivation. Also, operating room nurses can protect themselves against the harmful effects of burnout by strengthening interpersonal skills, stress management skills, having adequate rest and following ergonomic principles. It is suggested that studies are conducted to discover ways to prevent and coping with burnout in operating room nurses and also to evaluate different strategies in this field.

## Data availability statement

The original contributions presented in this study are included in the article/supplementary material, further inquiries can be directed to the corresponding authors.

## Author contributions

ET, AZ, SB-V, and RL involved in the study conception and design and contributed to the data collection and analysis. ET and SB-V drafted the manuscript. AZ, SB-V, and RL provided critical feedback on the study, qualitative analysis, and supervised the work. All authors contributed to the article and approved the submitted version.
